# Differentiation of Alzheimer's disease from other neurodegenerative disorders using chemiluminescence immunoassays measuring cerebrospinal fluid biomarkers

**DOI:** 10.3389/frdem.2024.1455619

**Published:** 2024-10-01

**Authors:** Philipp Arendt, Katharina Römpler, Britta Brix, Viola Borchardt-Lohölter, Mandy Busse, Stefan Busse

**Affiliations:** ^1^Institute for Experimental Immunology, Affiliated to EUROIMMUN Medizinische Labordiagnostika AG, Lübeck, Germany; ^2^Department for Experimental Obstetrics and Gynecology, Medical Faculty, Otto-von-Guericke-University of Magdeburg, Magdeburg, Germany; ^3^Department of Psychiatry and Psychotherapy, Medical Faculty University Hospital Magdeburg, Otto von Guericke University, Magdeburg, Germany

**Keywords:** Alzheimer's disease, ATN system, beta-amyloid, biomarker, chemiluminescence immunoassay, cerebrospinal fluid, neurodegenerative diseases, mild cognitive impairment

## Abstract

**Introduction:**

Prior research identified four neurochemical cerebrospinal fluid (CSF) biomarkers, Aβ1–42, Aβ1–40, tTau, and pTau(181), as core diagnostic markers for Alzheimer's disease (AD). Determination of AD biomarkers using immunoassays can support differential diagnosis of AD vs. several neuropsychiatric disorders, which is important because the respective treatment regimens differ. Results of biomarker determination can be classified according to the Amyloid/Tau/Neurodegeneration (ATN) system into profiles. Less is known about the clinical performance of chemiluminescence immunoassays (ChLIA) measuring specific biomarkers in CSF samples from patients suffering from neuropsychiatric impairments with various underlying causes.

**Methods:**

Chemiluminescence immunoassays (ChLIAs, EUROIMMUN) were used to determine Beta-Amyloid (1–40), Beta-Amyloid (1–42), Total-Tau, and pTau(181) concentrations in precharacterized cerebrospinal fluid (CSF) samples from 219 AD patients, 74 patients with mild cognitive impairment (MCI), and 220 disease control (DC) patients.

**Results:**

83.0% of AD patients had ATN profiles consistent with AD, whereas 85.5% of DC patients and 77.0% of MCI patients had profiles inconsistent with AD. AD patients showed significantly lower amyloid ratio Aβ1–42/Aβ1–40 (mean: 0.07) and significantly higher concentrations of tTau (mean: 901.6 pg/ml) and pTau(181) (mean: 129 pg/ml) compared to DC and MCI patients (all *p* values < 0.0071).

**Discussion:**

The ChLIAs effectively determined specific biomarkers and can support differential diagnostics of AD. Their quality was demonstrated in samples from 513 patients with cognitive impairments, representing a realistic mix of underlying causes for seeking treatment at a memory clinic.

## 1 Introduction

Dementia is a leading cause of disability and dependency among elderly people and is currently the seventh leading cause of death globally (WHO, 2023). Alzheimer's disease (AD) is the most common cause of dementia in old age (Gonzales et al., 2022) and involves loss of function of brain areas that control attention, thought, memory and language. AD usually begins with mild memory impairment and can lead to loss of ability to respond to the environment. The pathophysiology of AD involves formation of plaques and neurofibrillary tangles leading to degeneration of neurons and synapses. Extensive research has defined neurological hallmark symptoms of AD: overproduction and aggregation of extracellular beta-amyloid (Aβ) peptides Aβ1–40 and Aβ1–42 as well as accumulation and hyperphosphorylation of intracellular microtubule-associated Tau proteins in tissues of cortical and limbic brain areas (Chen et al., 2017; Naseri et al., 2019). The cerebrospinal fluid (CSF) of individuals who will develop AD exhibits significantly lowered Aβ1–42 concentrations already 5–10 years prior to the onset of cognitive impairments (Buchhave et al., 2012).

AD progression is grouped in three phases: preclinical, mild cognitive impairment (MCI), and dementia (Sperling et al., 2011).

Diagnosis of dementia is supported by results of neurological tests on attention, memory, problem solving, and other cognitive abilities, physical examination including assessment of motor skills, neuroimaging, and serological testing. Four neurochemical cerebrospinal fluid (CSF) biomarkers, Aβ1–42, Aβ1–40, Tau protein (tTau), and Tau phosphorylated at threonine 181 (pTau(181)), are currently used as core diagnostic markers for AD (Lewczuk et al., 2020). For differential diagnosis of AD, these biomarkers have an added value as they help to delineate AD from related disorders, mixed pathologies or atypical presentations (Bjerke and Engelborghs, 2018). The Aβ1–42 concentration in CSF of AD patients is inversely proportional to the amount of amyloid plaques and is ~50% lower than in cognitively healthy elderly individuals (Lewczuk et al., 2020). Although the Aβ1–40 concentration in CSF of AD patients shows no or only small changes, it is diagnostically relevant, because determination of the amyloid ratio Aβ1–42/Aβ1–40 is more reliable than Aβ1–42 as a single biomarker. The amyloid ratio is already significantly lower in patients in the preclinical or MCI stage (Bjerke and Engelborghs, 2018). Moreover, the negative influence of preanalytical factors (adsorption effects, degradation, effects caused by the material and size of the sample tubes, thawing-freezing cycles) and effects of inter-individual variability on the overall Aβ production are minimized by using the amyloid ratio (Vanderstichele et al., 2012; Hansson et al., 2019). The existing body of research on tTau suggests that it is an unspecific marker of neurodegeneration (Naseri et al., 2019; Jack et al., 2018). Significantly increased levels of pTau(181) in CSF can be found in AD patients in comparison to cognitively healthy individuals or patients with other neurodegenerative diseases such as dementia with Lewy bodies, Parkinson's disease or multiple system atrophy (Sperling et al., 2011; Blennow and Zetterberg, 2018). tTau and pTau(181) seem to be later markers of dementia, because altered amyloid metabolism precedes tau-related pathology and neuronal degeneration (Buchhave et al., 2012; Jack et al., 2019).

Dementias share symptoms with several neuropsychiatric disorders (Cummings, 2021; Ismail et al., 2016). For example, psychotic symptoms occur across a broad range of dementias including AD, fronto-temporal dementia (FTD), and dementia with Lewy bodies (LBD) and are associated with rapid disease progression and increased mortality (Fischer et al., 2020). It is challenging to differentiate psychotic symptoms as part of a prodromal dementia from psychotic symptoms in established or late-onset psychotic disorders (Fischer et al., 2020). Furthermore, geriatric depression is associated with both significant cognitive impairments and an increased risk for AD and it might be an etiological factor for AD (Linnemann and Lang, 2020). On the other hand, dementias are often associated with depressive symptoms (Brzezinska et al., 2020; Dafsari and Jessen, 2020; Kuring et al., 2018). Further shared neuropsychiatric symptoms are anxiety, changes in eating behavior, attention deficits, obsessions, compulsions, mood changes, fatigue, and headaches. Due to these overlaps in symptoms, a simple diagnostic approach to distinguish between those disorders is not available and differential diagnosis remains challenging. Fortunately, AD biomarkers have the potential to support differential diagnosis of AD vs. vascular dementia (VD), FTD, (geriatric) depression, substance abuse disorders, Parkinson's disease (PD), Creutzfeld-Jakob disease (CJD), LBD and others (Bouwman et al., 2022). Most studies on the performance of CSF biomarkers have focused on the comparison of AD patients to cognitively healthy elderly individuals. This selection bias regarding the inclusion criteria for the non-AD group does not represent realistic conditions (Bayart et al., 2019). In the day-to-day operations of a memory clinic, patients with diverse memory problems, cognitive impairment and depressive symptoms need to be differentiated from AD patients. Hence, it is important for a reliable differential diagnosis to measure AD biomarkers not only in cognitively healthy individuals or healthy elderly individuals, but rather in large diverse cohorts of patients with neurodegenerative and psychiatric disorders.

To describe a multidomain biomarker profile at the individual level, an unbiased categorization has been suggested in which AD biomarkers are divided into three main categories: A, T, and N (Jack et al., 2016). A stands for an amyloid biomarker (Aβ_1 − 42_ or Aβ_1 − 42_/Aβ_1 − 40_ ratio), T for tau pathology (pTau(181)) and N for neurodegeneration or neuronal injury (tTau). The ATN system is useful to detect incipient AD or mixed dementia (Eckerstrom et al., 2021). The different combinations of biomarkers (Delaby et al., 2021) are listed in Table 1. All markers can be quantified using imaging methods or by means of immunometric tests such as enzyme-linked immunosorbent assays (ELISA) or chemiluminescence immunoassays (ChLIA) using CSF samples. While ELISA was the reference method for measuring CSF biomarkers in the past, nowadays ChLIA is mostly used due to its faster processing. In the clinical realm, key requirements for the assays are high specificity and accuracy, as well as standardized protocols for simple processing and automatability. EUROIMMUN has developed four quantitative ChLIAs that provide robust and highly reproducible measurement of Aβ_1 − 40_, Aβ_1 − 42_, tTau, or pTau. Fully automated processing of the tests increases the efficiency and standardization of the analyses in the diagnostic laboratory.

**Table 1:** Categories of the amyloid/tau/neurodegeneration (ATN) system (Jack et al., 2016).

**Category**	**Meaning**
A–T–N–	“all normal,” in which amyloid (ratio Aβ_1 − 42_/Aβ_1 − 40_), pTau(181) and tTau are within reference range values, not consistent with AD
A+T+N+	“all pathological,” in which all biomarkers show pathological values, consistent with AD
A+T–N–	“amyloid,” in which only amyloid values are pathological, consistent with amyloidopathy
A–T–N+	“tTau,” in which only tTau values are pathological, inconsistent with AD, but may be consistent with other neurodegenerative diseases and/or neuronal damage
A+T–N+	“amyloid and tTau,” in which amyloid and tTau values are pathological, atypical profile, consistent with AD
A+T+N–	“amyloid and pTau(181),” in which amyloid and pTau(181) values are pathological, atypical profile, may be consistent with AD
A–T+N+	“pTau(181) and tTau,” in which values for both tau biomarkers are pathological, atypical profile, inconsistent with AD
A–T+N–	“pTau(181),” in which only pTau(181) values are pathological, atypical profile, inconsistent with AD

A: amyloid biomarker (Aβ1–42 or Aβ1–42/Aβ1–40 ratio), T: tau pathology (pTau(181)), N: neurodegeneration or neuronal injury (tTau). Different combinations of biomarkers describe profiles consistent or inconsistent with AD (Delaby et al., 2021).

There is little published data on the diagnostic accuracy of CSF biomarkers for diagnosis of AD using ChLIA (Bayart et al., 2019; Agnello et al., 2020). In this study, the biomarkers Aβ_1 − 40_, Aβ_1 − 42_, tTau, and pTau(181) were measured in CSF samples from the patients using four newly developed ChLIAs (commercially available from EUROIMMUN). Here, we evaluated clinical performance of the EUROIMMUN ChLIA for the first time and related the results to the ATN system. This study provides an authentic representation of the situation faced daily by clinicians in a memory clinic by including patients with AD or cognitive impairment, such as memory problems and mood disturbances, with various underlying causes.

## 2 Methods

### 2.1 Description of cohorts

All patients included in this study were retrospectively recruited from the geropsychiatric ward of the Department of Psychiatry and Psychotherapy, University Hospital Magdeburg, between 2012 and 2019. Patients were diagnosed by experienced psychiatrists, neurologists, and psychologists according to DSM-IV criteria, based on the anamnesis and results obtained by magnetic resonance imaging (MRI) and/or computed tomography (CT) of the brain, mini-mental state evaluation (MMSE), Montreal Cognitive Assessment (MoCa), CERAD test battery and CSF analysis performed at the University of Magdeburg. CSF analysis included determination of levels of Amyloid-β_1 − 40_, Amyloid-β_1 − 42_, total tau, and phospho-tau using INNOTEST immunoassays (Fujirebio). AD patients showed a typical clinical picture with slowly progressive course and memory impairment; neuropsychological tests showed cognitive deficits; MRI showed temporal and hippocampal atrophy; CSF showed corresponding pathologically altered values. MCI patients had lower scores in MMSE, but pathological MoCa; daily life could still be managed; CSF results were normal or pathologically altered. All other patients who had neither AD nor MCI were classified as DC.

This study utilized residual CSF samples following the completion of all diagnostic procedures. The patient data were anonymized. The study included 219 patients with AD (84 males, 135 females) and 74 patients with MCI (27 males, 47 females). The DC cohort comprised 220 patients (108 males, 112 females) suffering from AD-like symptoms such as FTD, VD, depression, schizophrenia, PD, alcohol-related dementia (ARD), LBD, CJD, delirium, schizo-affective disorder, delusional disorder, neuroborreliosis, panic disorder, personality disorder, normal pressure hydrocephalus (NPH), Wernicke encephalitis, cerebral angiopathy, and herpes encephalitis. Comorbidities with these diseases were considered. Patients with unclear dementia or mixed forms of dementia were excluded. The mean age of patients was 79.8 ± 7.3 years [range: (59, 95)], 75.4 ± 8.1 years [range: (55, 97)], and 72.4 ± 10.3 years [range: (34, 94)] in the AD, MCI, and DC cohorts, respectively.

### 2.2 Description of chemiluminescence immunoassays

Biomarker concentrations were determined using the Beta-Amyloid (1–40), Beta-Amyloid (1–42), Total-Tau, and pTau(181) ChLIAs (EUROIMMUN Medizinische Labordiagnostika AG, Luebeck, Germany) according to the manufacturer's instructions and as described elsewhere (Römpler et al., 2024). The Alzheimer ChLIAs were performed fully automated on the random-access device IDS-i10 (Immunodiagnostic Systems) in Lübeck by laboratory personnel blinded to results of both other diagnostic measures and the final diagnosis. The cut-offs of 741, 508, and 58.2 pg/ml were used for the evaluation of Aβ1–42, tTau, and pTau(181), respectively, as well as of 0.093 for the ratio Aβ1–42/ Aβ1–40.

### 2.3 ATN biomarker profiles

To relate the results to the ATN system (Jack et al., 2016; Delaby et al., 2021), the amyloid ratio Aβ1–42/Aβ1–40 was defined as A marker, p-Tau(181) as T marker, and tTau as N marker. Additionally, the ratios Aβ1–42/tTau and Aβ1–42/pTau(181) were reported as previous research has found that AD patients had reduced values for these measures (Agnello et al., 2020; Leitão et al., 2019).

### 2.4 Statistical analysis

Statistical analyses were performed using MATLAB R2019a. Lillefors tests indicated that biomarker profiles were not normally distributed but skewed. Kruskal–Wallis tests were performed to test for effects of diagnostic group (AD, MCI, DC) on biomarker value. Multiple comparison testing using the Mann–Whitney *U*-tests were performed to test for differences between two diagnostic groups. Bonferroni correction was applied to correct for multiple testing of seven biomarkers. *p*-values < 0.0071 were considered statistically significant. The standardized test statistic of the corresponding test and the number of cases were used to calculate the effect size r for the difference between two medians (i.e., suitable for the Mann–Whitney *U*-test). An r value below 0.3 is considered a small effect, between 0.3 and 0.5 as medium and values >0.5 as strong effects.

## 3 Results

### 3.1 Biomarker concentrations

The Kruskal–Wallis tests rejected the null hypothesis that all three data samples come from the same distribution (α = 0.05, df = 512, Table 2).

**Table 2:** Mean concentration ± standard deviation (SD) of biomarkers and mean ratios ± SD of Alzheimer's disease (AD), disease control (DC) patients, and patients with mild cognitive impairment (MCI).

**Biomarker**	**AD**	**DC**	**MCI**	**Kruskal–Wallis test**		**Mann–Whitney** ***U*****-tests**					
	***n*** = **219**	***n*** = **220**	***n*** = **74**	***n*** = **513**		**AD vs. DC**			**AD vs. MCI**		
	**Mean (**±**SD)**			***X** ^2^*	***p***	***U***	***p***	**r**	***U***	***p***	**r**
Aβ_1 − 40_	7,814.8 (2,532.0)	6,767.0 (2,198.4)	7,285.9 (2,432.1)	18.6	9.3 × 10^−5^	4.3	1.6 × 10^−5^	0.22	1.5	0.12	0.09
Aβ_1 − 42_	578.2 (264.3)	884.9 (331.3)	883.8 (339.4)	117.7	2.7 × 10^−26^	−10.3	9.9 × 10^−25^	−0.46	−6.9	4.1 × 10^−12^	−0.43
Aβ_1 − 42_/Aβ_1 − 40_	0.07 (0.02)	0.13 (0.03)	0.12 (0.03)	252.6	1.4 × 10^−55^	−15.3	9.3 × 10^−53^	−0.74	−9.4	4.1 × 10^−21^	−0.64
tTau	901.6 (500.6)	485.9 (148.8)	450.0 (506.8)	240.5	5.8 × 10^−53^	14.7	4.9 × 10^−49^	0.18	9.7	2.0 × 10^−22^	0.36
pTau(181)	129.0 (69.4)	37.3 (23.0)	41.3 (25.2)	303.0	1.6 × 10^−66^	16.4	1.3 × 10^−60^	0.66	11.2	5.6 × 10^−29^	0.53
Aβ_1 − 42_/tTau	0.79 (0.5)	2.8 (1.3)	2.6 (1.2)	275.1	1.8 × 10^−60^	−15.7	2.8 × 10^−55^	−0.71	−10.6	3.5 × 10^−26^	−0.72
Aβ_1 − 42_/pTau(181)	6.2 (6.2)	29.8 (14.2)	27.8 (14.2)	288.2	2.7 × 10^−63^	−10.9	6.5 × 10^−58^	−0.73	−10.9	1.8 × 10^−27^	−0.73

U: Mann–Whitney U-value; p: p-values of Mann–Whitney U-tests; r: effect size. A negative sign of r is irrelevant to the difference itself as it depends on which group is considered first.

**Figure 1:**
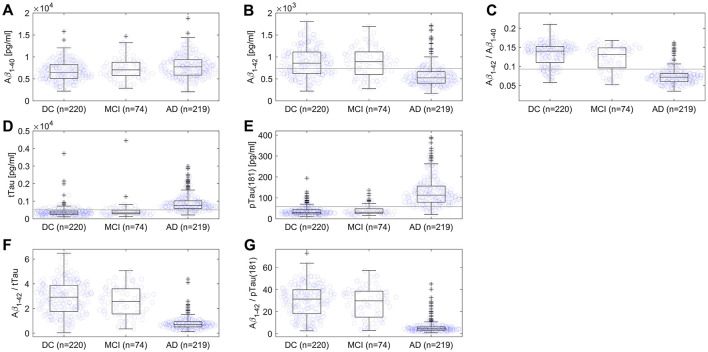
Scatterplots and boxplots comparing values of **(A)** Aβ1–40, **(B)** Aβ1–42, **(C)** ratio Aβ1–42/Aβ1–40, **(D)** tTau, **(E)** pTau(181), **(F)** ratio Aβ1–42/tTau, and **(G)** ratio Aβ1–42/pTau(181) for 219 Alzheimer's disease (AD) and 220 disease control (DC) patients, and 74 patients with mild cognitive impairment (MCI). On each box, the central mark indicates the median, and the bottom and top edges of the box indicate the 25th and 75th percentiles, respectively. The whiskers extend to the most extreme data points not considered outliers, and outliers are plotted as crosses. The gray line represents the assay's cut-off. One DC patient with Creutzfeldt-Jakob disease and one MCI patient were not displayed in **(D)** due to very high values of tTau.

In AD patients, significantly higher concentrations of Aβ1–40, tTau, and pTau(181) were found (all *p* < 0.001), while lower concentrations of Aβ1–42, as well as lower ratios of Aβ1–42/Aβ1–40, Aβ1–42/tTau, and Aβ1–42/pTau(181) were observed compared to DC patients and indicated by the respective U values (Figure 1, Table 2). Note that effect sizes were small for between-group differences in Aβ1–40 and tTau, medium for Aβ1–42, but strong for pTau(181), Aβ1–42/tTau, Aβ1–42/pTau(181), and Aβ1–42/Aβ1–40 (Table 2).

Compared to samples from patients with MCI, AD patient samples showed significantly (all *p* < 0.001) higher amounts of tTau and pTau(181), but significantly lower quantities of Aβ1–42 (Table 2). The ratios Aβ1–42/Aβ1–40, Aβ1–42/tTau and Aβ1–42/pTau(181) were significantly lower in AD patients (Figure 1, Table 2). For Aβ1–40, group differences between AD and MCI were not significantly different (*p* = 0.12). Effect sizes were small for between-group differences in Aβ1–40, medium for Aβ1–42 and tTau, but strong for pTau(181), Aβ1–42/tTau, Aβ1–42/pTau(181), and Aβ1–42/Aβ1–40 (Table 2).

**Figure 2:**
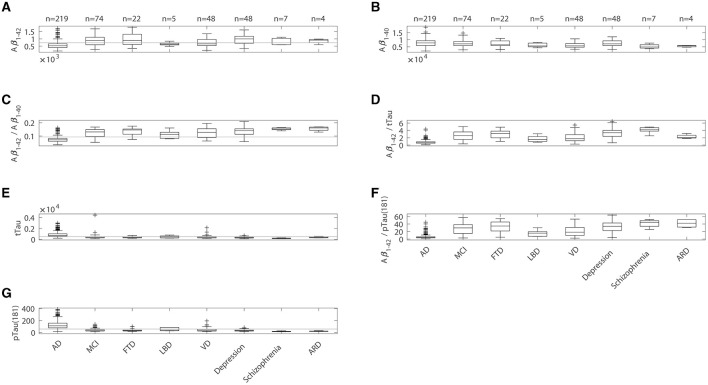
Boxplots comparing values of **(A)** Aβ1–42, **(B)** Aβ1–40, **(C)** ratio Aβ1–42/Aβ1–40, **(D)** ratio Aβ1–42/tTau, **(E)** tTau, **(F)** ratio Aβ1–42/pTau(181), and **(G)** pTau(181) determined in samples from patients with Alzheimer's disease (AD), mild cognitive impairment (MCI) and six patient groups among the disease control (DC) patients, such as fronto-temporal dementia (FTD), dementia with Lewy bodies (LBD), vascular dementia (VD), depression, schizophrenia and alcohol addiction (ARD). On each box, the central mark indicates the median, and the bottom and top edges of the box indicate the 25th and 75th percentiles, respectively. The whiskers extend to the most extreme data points not considered outliers, and outliers are plotted as crosses. The gray line represents the assay's cut-off.

For qualitative comparisons with AD and MCI patients, values for pTau(181), Aβ1–42/tTau, Aβ1–42/pTau(181), and Aβ1–42/Aβ1–40 of DC patients with FTD, LBD, VD, depression, schizophrenia, and ARD were plotted separately (Figure 2). Differences between patient groups can be observed by trend, but should be evaluated with caution, because the respective group sizes differ and do not allow robust statistical statements.

### 3.2 ATN profiles

**Figure 3:**
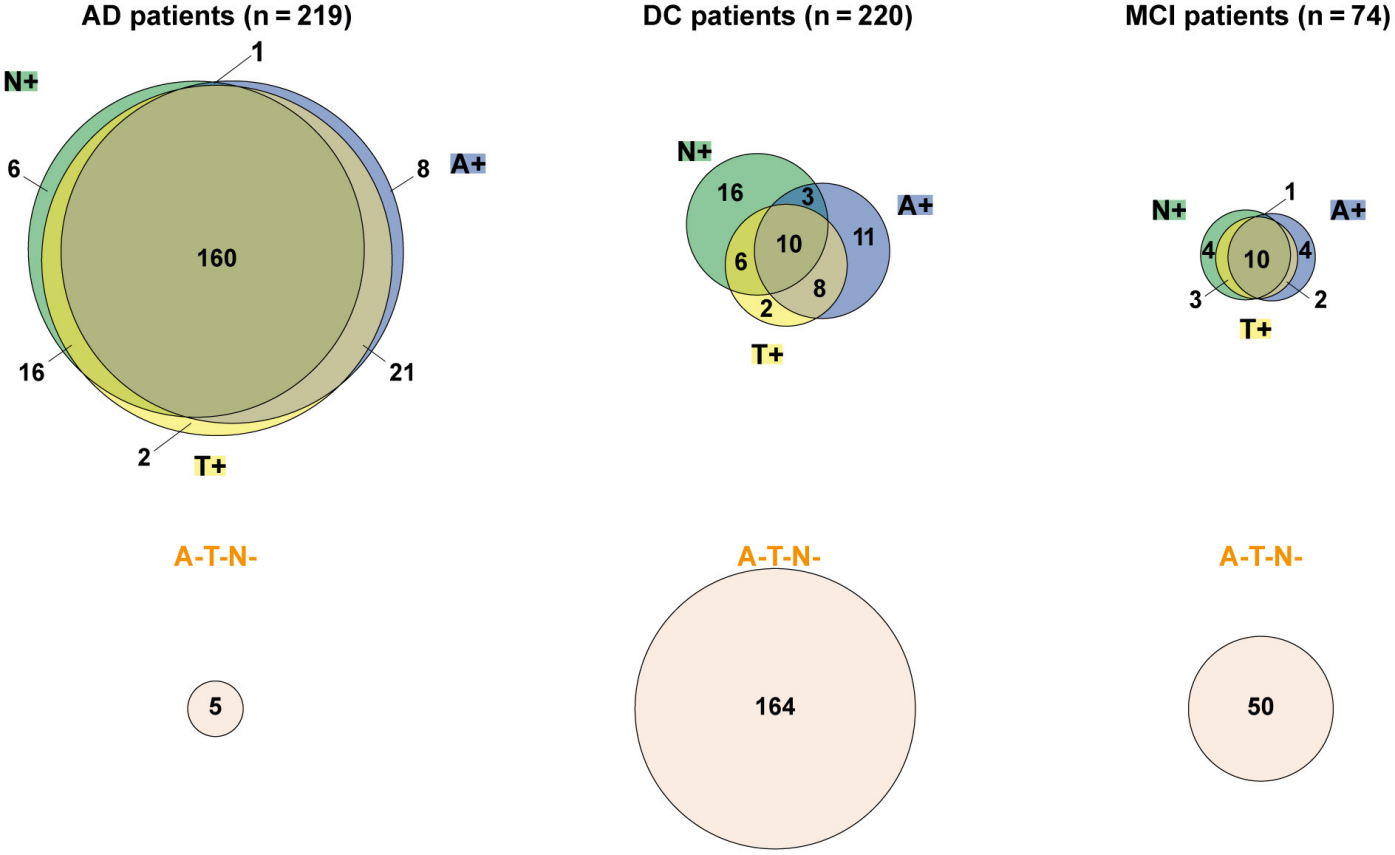
Area-proportional Venn diagrams visualizing proportional set relationships of biomarker profiles of Alzheimer's disease (AD) vs. disease control (DC) and patients with mild cognitive impairment (MCI) according to Jack et al. (2016) and Delaby et al. (2021), created using BioVenn (Hulsen et al., 2008).

83.1% of AD patients had ATN profiles (Table 1) consistent with AD (A+T+N+: 73.1%, A+T+N–: 9.6%, A+T–N+: 0.5%, Table 3, Figure 3). ATN profiles inconsistent with AD were observed in 77.0% of MCI patients (A–T–N–: 67.6%, A–T–N+: 5.4%, A–T+N–: 0%, A–T+N+: 4.1%) and in 85.5% of DC patients (A–T–N–: 74.6%, A–T–N+: 7.3%, A–T+N–: 0.9%, A–T+N+: 2.7%, Table 3, Figure 3).

**Table 3:** Distributions of biomarker profiles of Alzheimer's disease (AD) patients and disease control (DC) patients according to Jack et al. (2016) and Delaby et al. (2021).

**Biomarker profile**	**AD (*n* = 219)**	**DC (*n* = 220)**	**MCI (*n* = 74)**
A– T– N–	5 (2.3%)	164 (74.6%)	50 (67.6%)
A+ T– N–	8 (3.7%)	11 (5.0%)	4 (5.4%)
A+ T+ N–	21 (9.6%)	8 (3.6%)	2 (2.7%)
A+ T– N+	1 (0.5%)	3 (1.4%)	1 (1.4%)
A– T+ N+	16 (7.3%)	6 (2.7%)	3 (4.1%)
A– T– N+	6 (2.7%)	16 (7.3%)	4 (5.4%)
A– T+ N–	2 (0.9%)	2 (0.9%)	0 (0.0%)
A+ T+ N+	160 (73.1%)	10 (4.6%)	10 (13.5%)
A+	190 (86.8%)	32 (14.6%)	17 (23.0%)
T+	199 (90.9%)	26 (11.8%)	15 (20.3%)
N+	183 (83.6%)	35 (15.9%)	18 (24.3%)

One CJD patient with the biomarker profile A–T+N+ had an extremely high value for tTau. This is in accordance with a comment on this biomarker profile by Delaby et al. (2021) stating that very high levels of tTau (close to or above the upper detection limit of the assay) speak in favor of Creutzfeld-Jakob disease, if other causes of major neuronal injury are excluded. The patient was defined as an outlier and not displayed in Figure 1D.

## 4 Discussion

The present study demonstrated the clinical validity of serological testing using ChLIAs based on analysis of CSF samples from 513 patients suffering from neuropsychiatric impairments with various underlying causes. The inclusion of AD, MCI, and DC patients allowed a realistic reflection of the daily situation clinicians of a memory clinic face. The challenging task of differentiating AD from neuropsychiatric disorders with similar symptoms can be supported by investigation of biomarker concentrations in CSF samples and categorization of results according to the ATN system.

AD patients showed significantly higher amounts of tTau and pTau(181), but significantly lower amounts of Aβ1–42, amyloid ratio Aβ1–42/Aβ1–40, Aβ1–42/tTau, and Aβ1–42/pTau(181) compared to both DC and MCI patients (Figure 1). Effect sizes of between-group differences in pTau(181), Aβ1–42/tTau, Aβ1–42/ pTau(181), and Aβ1–42/Aβ1–40 were strong and are therefore useful to support serological diagnostics (Table 2).

The distributions of biomarker profiles clearly differed between AD vs. DC and MCI patients (Figure 3). Biomarker profiles indicative of AD were found in 83.1% of CSF samples from AD patients, whereas 85.5 and 77.0% of CSF samples from DC and MCI patients, respectively, had biomarker values within reference range values, i.e., inconsistent with AD or amyloid pathology (Table 3). Compared to DC patients, more MCI patients had biomarker profiles indicative of pathological values for all three biomarkers (Table 3). The quantification of CSF core biomarkers in AD and DC patients was consistent with previous literature (Lewczuk et al., 2020). This research confirmed that AD biomarker profiles measured using the Alzheimer ChLIAs can support differential diagnosis of neurodegenerative diseases such as AD and related disorders with neuropsychiatric symptoms.

### 4.1 Limitations

Importantly, concentrations of CSF biomarkers do not constitute proof of presence or absence of a disease, but serve to support the diagnosis made by the clinician. Especially if a patient sample presented with biomarker profiles hinting toward an amyloid pathology or an atypical biochemical profile, the result should be interpreted in conjunction with results of other diagnostic methods such as neuroimaging and further clinical findings.

The study does not include follow-up analyses of the same patients or a neuropathological confirmation of AD diagnosis. The cohorts were not balanced for age and sex, since the aim was for the study cohorts to reflect a realistic mixture of middle-aged and elderly patients seeking treatment for cognitive impairment such as memory problems with various underlying causes. Influences of both age and sex on dementia and Alzheimer's disease have been addressed in previous research (Gonzales et al., 2022; Carter et al., 2012; GBD 2016 Dementia Collaborators, 2019). Data for the current study were acquired in only one clinic and future studies should include multicentric data to overcome potential effects of site or region. However, few studies have investigated cohorts as large as the current one. In most studies on the same topic, AD patients are compared to cognitively healthy elderly individuals, MCI patients or patients with lumbar puncture for reasons other than diagnosis of a neurodegenerative disease (Leitão et al., 2019; Niemantsverdriet et al., 2017). For research purposes, it is reasonable to assess biomarker levels in healthy subjects, MCI, and AD patients to shed light on Alzheimer's pathology. But to uncover measurable distinctions between AD, other dementias, and related neuropsychiatric disorders, it is necessary to study a realistic mixture of patients of a memory clinic. Therefore, the inclusion of patients with related diseases reflects an authentic patient population and fits the current study's aim.

It is not the task of this paper to examine methodological validity or analytical performance of the new ChLIA, since those have been demonstrated during their certification processes and have been published (Römpler et al., 2024).

### 4.2 Outlook

Differential diagnostics of dementias is challenging and often lengthy, which results in an economic burden regarding costs for medical care. To reduce time to diagnosis, an early use of reliable biomarkers is recommended. Biofluid biomarker screening is especially advantageous in regions of the world where access to highly specialized and expensive diagnostic instruments such as positron emission tomography or magnetic resonance imaging is limited, and trained staff is not available. Biomarker determination presents with the possibility of screening for several pathologies in parallel, which accelerates diagnosis. Differentiation of AD from other forms of dementia is important because the respective treatment regimens differ. A timely diagnosis of AD is relevant for families to plan appropriate care for the affected family member. In future, serologically derived biomarker results could be used for risk profiling according to the ATN system in individuals with subjective cognitive decline presenting at a memory clinic (Ebenau et al., 2020). Promising results were shown for very early detection of AD-related biomarkers using Raman spectroscopy techniques in CSF and serum samples (Polykretis et al., 2022).

## 5 Conclusions

Findings of the present study demonstrated the clinical validity of the Beta-Amyloid (1–40) ChLIA, Beta-Amyloid (1–42) ChLIA, Total-Tau ChLIA, and pTau(181) ChLIA from EUROIMMUN. They can be used in daily clinical routine to support diagnosis of AD as well as differentiation of amyloid pathologies, AD and other diseases involving cognitive deficits and memory problems.

## Data Availability

The raw data supporting the conclusions of this article will be made available by the authors, without undue reservation.
