# Corrigendum: Differentiation of Alzheimer's disease from other neurodegenerative disorders using chemiluminescence immunoassays measuring cerebrospinal fluid biomarkers

**DOI:** 10.3389/frdem.2025.1568275

**Published:** 2025-02-17

**Authors:** Philipp Arendt, Katharina Römpler, Britta Brix, Viola Borchardt-Lohölter, Mandy Busse, Stefan Busse

**Affiliations:** ^1^Institute for Experimental Immunology, Affiliated to EUROIMMUN Medizinische Labordiagnostika AG, Lübeck, Germany; ^2^Department for Experimental Obstetrics and Gynecology, Medical Faculty, Otto-von-Guericke-University of Magdeburg, Magdeburg, Germany; ^3^Department of Psychiatry and Psychotherapy, Medical Faculty University Hospital Magdeburg, Otto von Guericke University, Magdeburg, Germany

**Keywords:** Alzheimer's disease, ATN system, beta-amyloid, biomarker, chemiluminescence immunoassay, cerebrospinal fluid, neurodegenerative diseases, mild cognitive impairment

In the published article, there was an error in Figure 1D as published, where the labels of the x-axis in were incorrectly written as “AD (*n* = 220), DC (*n* = 218), MCI (*n* = 73).” The correct labels are “DC (*n* = 220), MCI (*n* = 74), AD (*n* = 219).”

The corrected Figure 1 and its caption appear below.

**Figure 1:**
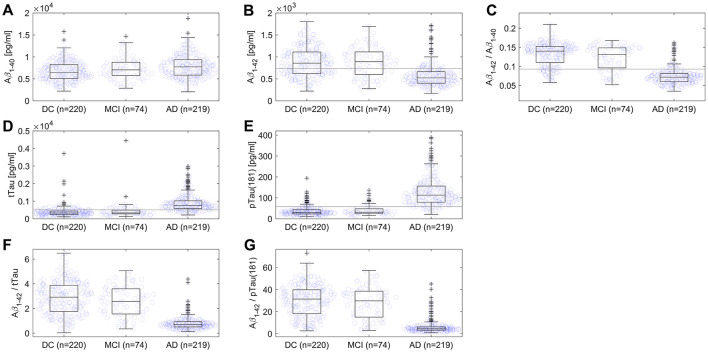
Scatterplots and boxplots comparing values of **(A)** Aβ1–40, **(B)** Aβ1–42, **(C)** ratio Aβ1–42/Aβ1–40, **(D)** tTau, **(E)** pTau(181), **(F)** ratio Aβ1–42/tTau, and **(G)** ratio Aβ1–42/pTau(181) for 219 Alzheimer's disease (AD) and 220 disease control (DC) patients, and 74 patients with mild cognitive impairment (MCI). On each box, the central mark indicates the median, and the bottom and top edges of the box indicate the 25th and 75th percentiles, respectively. The whiskers extend to the most extreme data points not considered outliers, and outliers are plotted as crosses. The gray line represents the assay's cut-off. One DC patient with Creutzfeldt-Jakob disease and one MCI patient were not displayed in **(D)** due to very high values of tTau.

The authors apologize for this error and state that this does not change the scientific conclusions of the article in any way. The original article has been updated.

